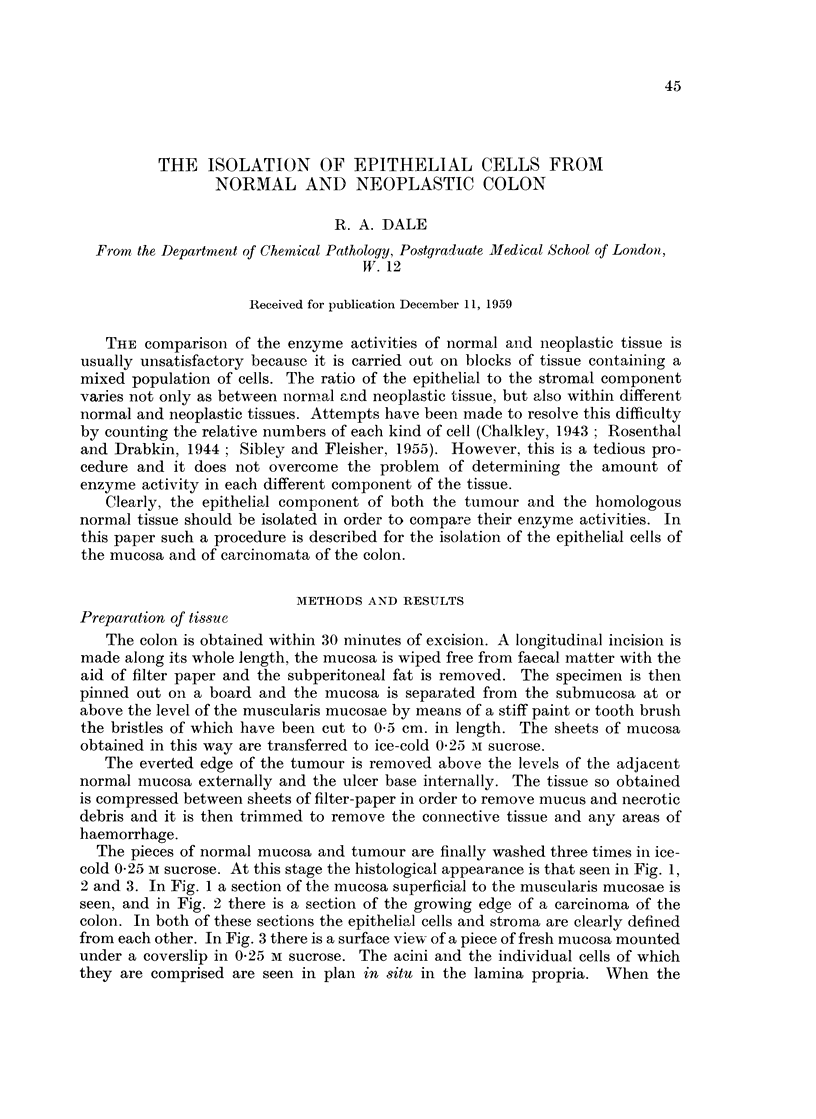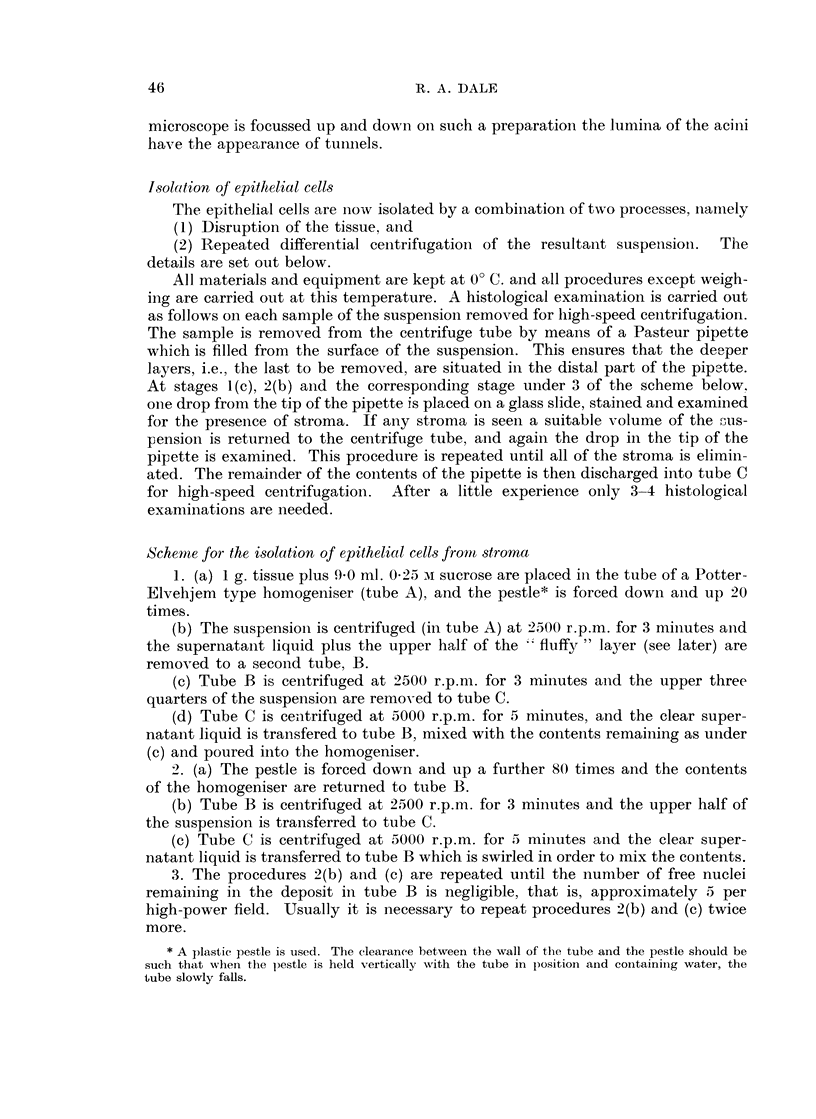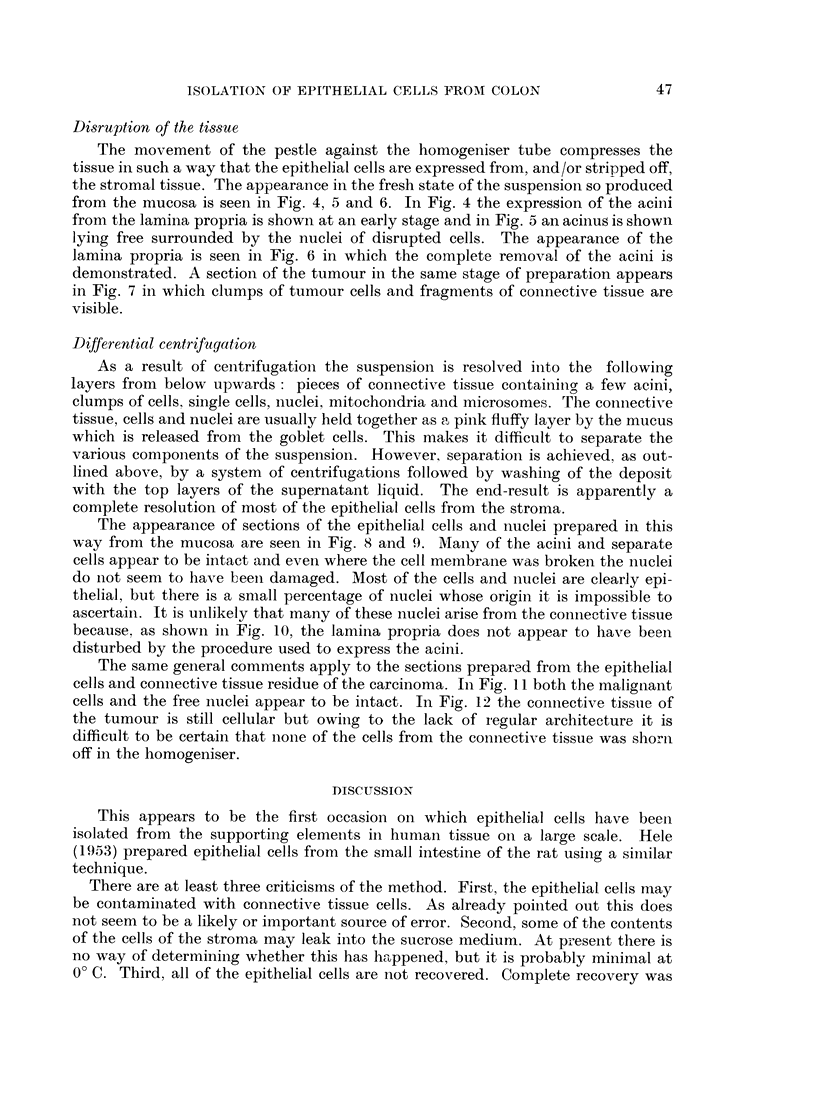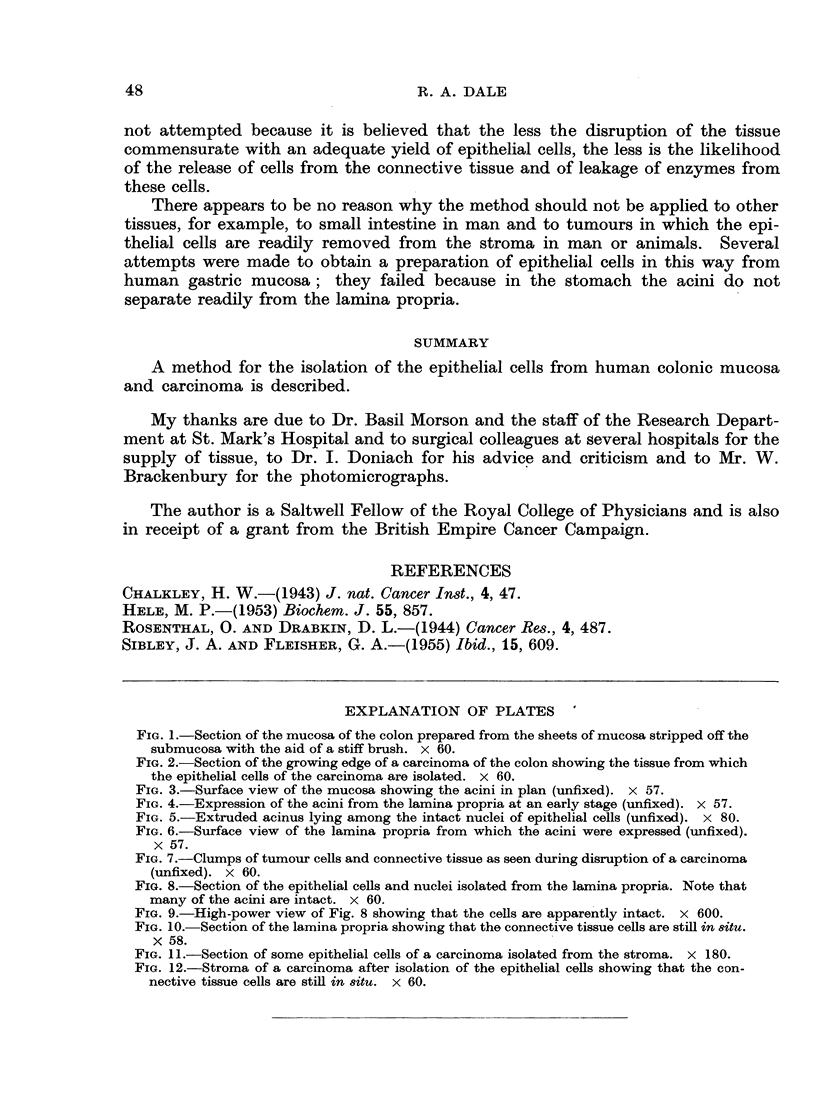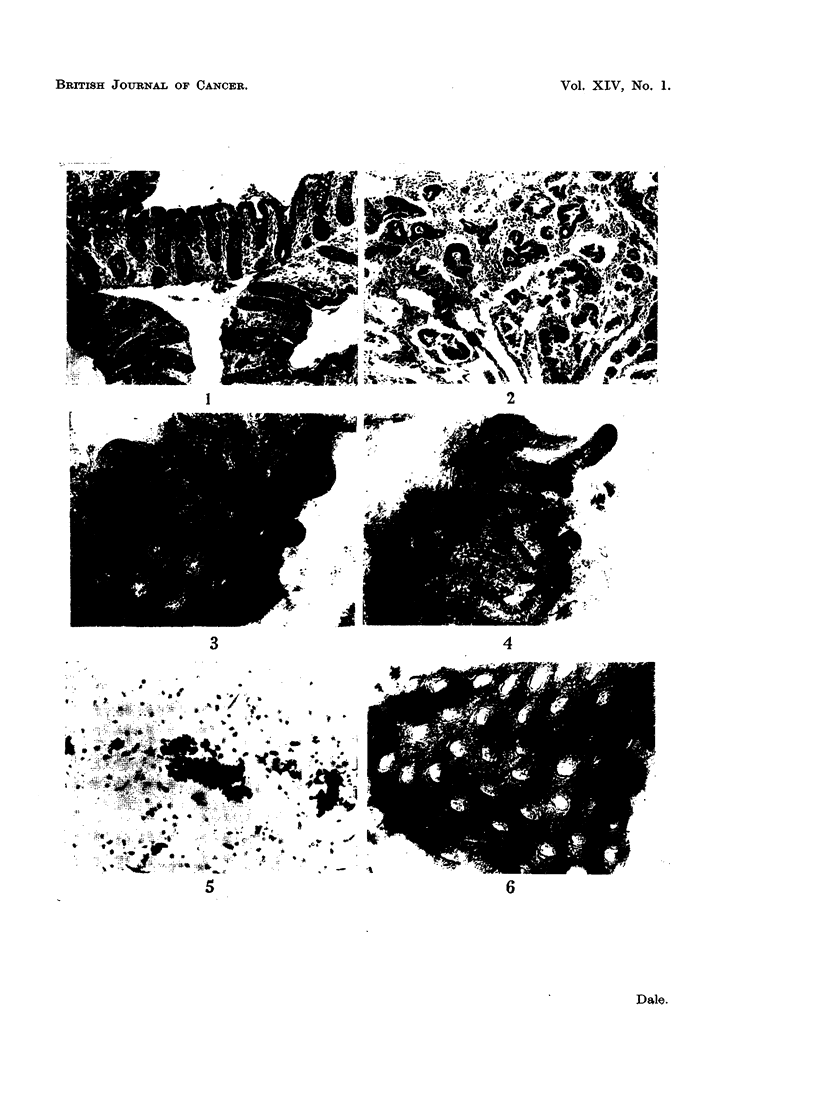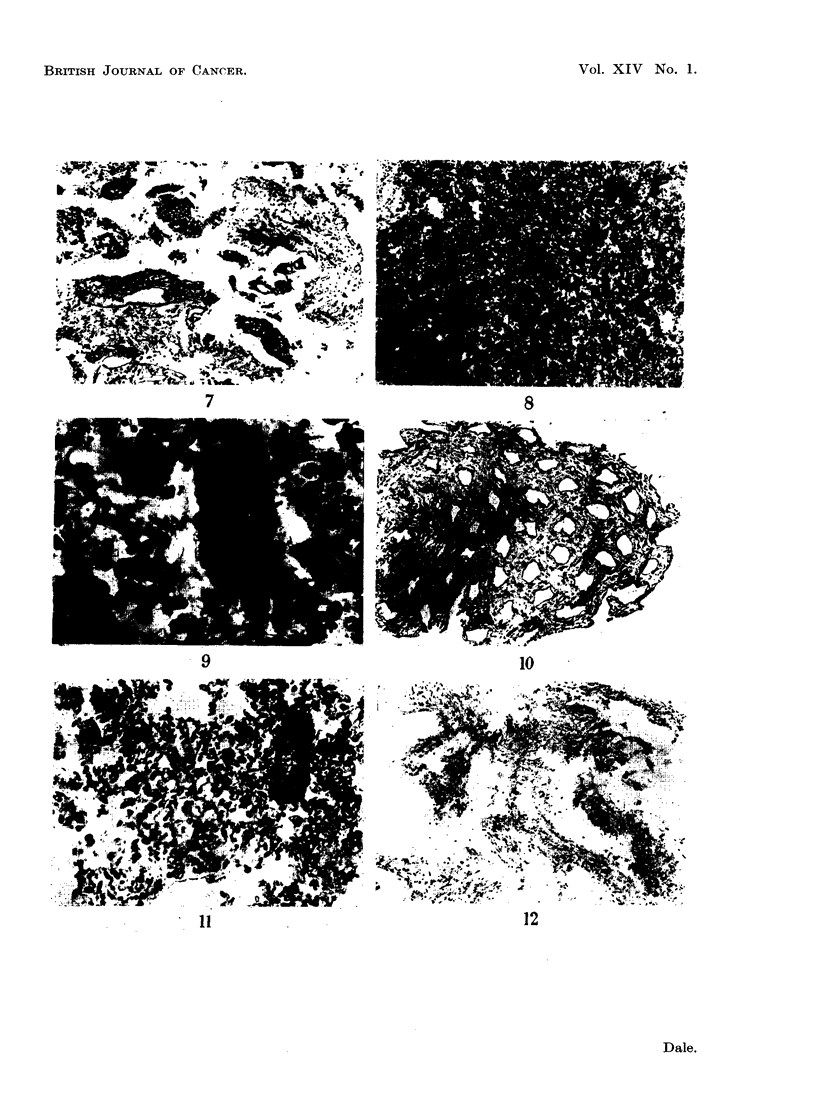# The Isolation of Epithelial Cells from Normal and Neoplastic Colon

**DOI:** 10.1038/bjc.1960.5

**Published:** 1960-03

**Authors:** R. A. Dale

## Abstract

**Images:**


### THE ISOLATION OF EPITHELIAL CELLS FROM NORMAL AND NEOPLASTIC COLON

R. A. DALE

*From the Department of Chemical Pathology, Postgraduate Medical School of London, W. 12*

Received for publication December 11, 1959

THE comparison of the enzyme activities of normal and neoplastic tissue is usually unsatisfactory because it is carried out on blocks of tissue containing a mixed population of cells. The ratio of the epithelial to the stromal component varies not only as between normal and neoplastic tissue, but also within different normal and neoplastic tissues. Attempts have been made to resolve this difficulty by counting the relative numbers of each kind of cell (Chalkley, 1943 ; Rosenthal and Drabkin, 1944 ; Sibley and Fleisher, 1955). However, this is a tedious procedure and it does not overcome the problem of determining the amount of enzyme activity in each different component of the tissue.

Clearly, the epithelial component of both the tumour and the homologous normal tissue should be isolated in order to compare their enzyme activities. In this paper such a procedure is described for the isolation of the epithelial cells of the mucosa and of carcinomata of the colon.

### METHODS AND RESULTS

### *Preparation of tissue*

The colon is obtained within 30 minutes of excision. A longitudinal incision is made along its whole length, the mucosa is wiped free from faecal matter with the aid of filter paper and the subperitoneal fat is removed. The specimen is then pinned out on a board and the mucosa is separated from the submucosa at or above the level of the muscularis mucosae by means of a stiff paint or tooth brush the bristles of which have been cut to 0·5 cm. in length. The sheets of mucosa obtained in this way are transferred to ice-cold 0·25 M sucrose.

The everted edge of the tumour is removed above the levels of the adjacent normal mucosa externally and the ulcer base internally. The tissue so obtained is compressed between sheets of filter-paper in order to remove mucus and necrotic debris and it is then trimmed to remove the connective tissue and any areas of haemorrhage.

The pieces of normal mucosa and tumour are finally washed three times in ice-cold 0·25 M sucrose. At this stage the histological appearance is that seen in Fig. 1, 2 and 3. In Fig. 1 a section of the mucosa superficial to the muscularis mucosae is seen, and in Fig. 2 there is a section of the growing edge of a carcinoma of the colon. In both of these sections the epithelial cells and stroma are clearly defined from each other. In Fig. 3 there is a surface view of a piece of fresh mucosa mounted under a coverslip in 0·25 M sucrose. The acini and the individual cells of which they are comprised are seen in plan *in situ* in the lamina propria. When the microscope is focussed up and down on such a preparation the lumina of the acini have the appearance of tunnels.

*Isolation of epithelial cells*

The epithelial cells are now isolated by a combination of two processes, namely
(1) Disruption of the tissue, and
(2) Repeated differential centrifugation of the resultant suspension. The details are set out below.

All materials and equipment are kept at 0° C. and all procedures except weighing are carried out at this temperature. A histological examination is carried out as follows on each sample of the suspension removed for high-speed centrifugation. The sample is removed from the centrifuge tube by means of a Pasteur pipette which is filled from the surface of the suspension. This ensures that the deeper layers, i.e., the last to be removed, are situated in the distal part of the pipette. At stages 1(c), 2(b) and the corresponding stage under 3 of the scheme below, one drop from the tip of the pipette is placed on a glass slide, stained and examined for the presence of stroma. If any stroma is seen a suitable volume of the suspension is returned to the centrifuge tube, and again the drop in the tip of the pipette is examined. This procedure is repeated until all of the stroma is eliminated. The remainder of the contents of the pipette is then discharged into tube C for high-speed centrifugation. After a little experience only 3–4 histological examinations are needed.

*Scheme for the isolation of epithelial cells from stroma*

1. (a) 1 g. tissue plus 9·0 ml. 0·25 M sucrose are placed in the tube of a Potter-Elvehjem type homogeniser (tube A), and the pestle* is forced down and up 20 times.

(b) The suspension is centrifuged (in tube A) at 2500 r.p.m. for 3 minutes and the supernatant liquid plus the upper half of the " fluffy " layer (see later) are removed to a second tube, B.

(c) Tube B is centrifuged at 2500 r.p.m. for 3 minutes and the upper three quarters of the suspension are removed to tube C.

(d) Tube C is centrifuged at 5000 r.p.m. for 5 minutes, and the clear supernatant liquid is transfered to tube B, mixed with the contents remaining as under (c) and poured into the homogeniser.

2. (a) The pestle is forced down and up a further 80 times and the contents of the homogeniser are returned to tube B.

(b) Tube B is centrifuged at 2500 r.p.m. for 3 minutes and the upper half of the suspension is transferred to tube C.

(c) Tube C is centrifuged at 5000 r.p.m. for 5 minutes and the clear supernatant liquid is transferred to tube B which is swirled in order to mix the contents.

3. The procedures 2(b) and (c) are repeated until the number of free nuclei remaining in the deposit in tube B is negligible, that is, approximately 5 per high-power field. Usually it is necessary to repeat procedures 2(b) and (c) twice more.

* A plastic pestle is used. The clearance between the wall of the tube and the pestle should be such that when the pestle is held vertically with the tube in position and containing water, the tube slowly falls.

*Disruption of the tissue*

The movement of the pestle against the homogeniser tube compresses the tissue in such a way that the epithelial cells are expressed from, and/or stripped off, the stromal tissue. The appearance in the fresh state of the suspension so produced from the mucosa is seen in Fig. 4, 5 and 6. In Fig. 4 the expression of the acini from the lamina propria is shown at an early stage and in Fig. 5 an acinus is shown lying free surrounded by the nuclei of disrupted cells. The appearance of the lamina propria is seen in Fig. 6 in which the complete removal of the acini is demonstrated. A section of the tumour in the same stage of preparation appears in Fig. 7 in which clumps of tumour cells and fragments of connective tissue are visible.

*Differential centrifugation*

As a result of centrifugation the suspension is resolved into the following layers from below upwards : pieces of connective tissue containing a few acini, clumps of cells, single cells, nuclei, mitochondria and microsomes. The connective tissue, cells and nuclei are usually held together as a pink fluffy layer by the mucus which is released from the goblet cells. This makes it difficult to separate the various components of the suspension. However, separation is achieved, as outlined above, by a system of centrifugations followed by washing of the deposit with the top layers of the supernatant liquid. The end-result is apparently a complete resolution of most of the epithelial cells from the stroma.

The appearance of sections of the epithelial cells and nuclei prepared in this way from the mucosa are seen in Fig. 8 and 9. Many of the acini and separate cells appear to be intact and even where the cell membrane was broken the nuclei do not seem to have been damaged. Most of the cells and nuclei are clearly epithelial, but there is a small percentage of nuclei whose origin it is impossible to ascertain. It is unlikely that many of these nuclei arise from the connective tissue because, as shown in Fig. 10, the lamina propria does not appear to have been disturbed by the procedure used to express the acini.

The same general comments apply to the sections prepared from the epithelial cells and connective tissue residue of the carcinoma. In Fig. 11 both the malignant cells and the free nuclei appear to be intact. In Fig. 12 the connective tissue of the tumour is still cellular but owing to the lack of regular architecture it is difficult to be certain that none of the cells from the connective tissue was shorn off in the homogeniser.

### DISCUSSION

This appears to be the first occasion on which epithelial cells have been isolated from the supporting elements in human tissue on a large scale. Hele (1953) prepared epithelial cells from the small intestine of the rat using a similar technique.

There are at least three criticisms of the method. First, the epithelial cells may be contaminated with connective tissue cells. As already pointed out this does not seem to be a likely or important source of error. Second, some of the contents of the cells of the stroma may leak into the sucrose medium. At present there is no way of determining whether this has happened, but it is probably minimal at 0° C. Third, all of the epithelial cells are not recovered. Complete recovery was not attempted because it is believed that the less the disruption of the tissue commensurate with an adequate yield of epithelial cells, the less is the likelihood of the release of cells from the connective tissue and of leakage of enzymes from these cells.

There appears to be no reason why the method should not be applied to other tissues, for example, to small intestine in man and to tumours in which the epithelial cells are readily removed from the stroma in man or animals. Several attempts were made to obtain a preparation of epithelial cells in this way from human gastric mucosa ; they failed because in the stomach the acini do not separate readily from the lamina propria.

### SUMMARY

A method for the isolation of the epithelial cells from human colonic mucosa and carcinoma is described.

My thanks are due to Dr. Basil Morson and the staff of the Research Department at St. Mark's Hospital and to surgical colleagues at several hospitals for the supply of tissue, to Dr. I. Doniach for his advice and criticism and to Mr. W. Brackenbury for the photomicrographs.

The author is a Saltwell Fellow of the Royal College of Physicians and is also in receipt of a grant from the British Empire Cancer Campaign.

### REFERENCES

CHALKLEY, H. W.—(1943) *J. nat. Cancer Inst.*, **4**, 47.
HELE, M. P.—(1953) *Biochem. J.* **55**, 857.
ROSENTHAL, O. AND DRABKIN, D. L.—(1944) *Cancer Res.*, **4**, 487.
SIBLEY, J. A. AND FLEISHER, G. A.—(1955) *Ibid.*, **15**, 609.

### EXPLANATION OF PLATES

FIG. 1.—Section of the mucosa of the colon prepared from the sheets of mucosa stripped off the submucosa with the aid of a stiff brush. × 60.
FIG. 2.—Section of the growing edge of a carcinoma of the colon showing the tissue from which the epithelial cells of the carcinoma are isolated. × 60.
FIG. 3.—Surface view of the mucosa showing the acini in plan (unfixed). × 57.
FIG. 4.—Expression of the acini from the lamina propria at an early stage (unfixed). × 57.
FIG. 5.—Extruded acinus lying among the intact nuclei of epithelial cells (unfixed). × 80.
FIG. 6.—Surface view of the lamina propria from which the acini were expressed (unfixed). × 57.
FIG. 7.—Clumps of tumour cells and connective tissue as seen during disruption of a carcinoma (unfixed). × 60.
FIG. 8.—Section of the epithelial cells and nuclei isolated from the lamina propria. Note that many of the acini are intact. × 60.
FIG. 9.—High-power view of Fig. 8 showing that the cells are apparently intact. × 600.
FIG. 10.—Section of the lamina propria showing that the connective tissue cells are still *in situ*. × 58.
FIG. 11.—Section of some epithelial cells of a carcinoma isolated from the stroma. × 180.
FIG. 12.—Stroma of a carcinoma after isolation of the epithelial cells showing that the connective tissue cells are still *in situ*. × 60.